# How do patients choose between obesity medications: A thematic analysis

**DOI:** 10.1016/j.obpill.2025.100187

**Published:** 2025-06-16

**Authors:** Alvin Mondoh, Hilary Craig, Michael Crotty, Francisca Contreras, Carel W. le Roux

**Affiliations:** a Diabetes Complications Research Centre, Conway Institute of Biomedical and Biomolecular Research, University College Dublin, Dublin 4, Ireland; b UCD School of Medicine, University College Dublin, Dublin 4, Ireland; c My Best Weight Clinic, Dublin, Ireland

**Keywords:** Obesity medication, Effectiveness, Type 2 diabetes, Obesity, Safety, Weight loss

## Abstract

**Introduction:**

The rising prevalence of obesity is of particular concern due to its association with a range of serious complications, including type 2 diabetes mellitus, cardiovascular diseases, and certain types of cancer. Obesity medications can control the disease but it is unclear how patients choose which medication to use.

**Methods:**

A qualitative thematic analysis was conducted to investigate how patients select between obesity medications. Fifteen treatment naive adults with a body mass index ≥27 kg/m^2^ with at least one weight-related complications were recruited.

**Results:**

The 5 major themes depicting how patients make selections included 1) Effectiveness of medication, 2) Information to make decisions, 3) Safety of medications, 4) Practicality and 5) Individual Strategies and Community Supports in Obesity Management. Safety concerns of side effects and long-term risks were perceived major barriers to initiating or adhering to pharmacotherapy.

**Conclusion:**

In a situation where the medications are described as being free and readily available, patient preferences for obesity medications are shaped by treatment efficacy, safety, information provided by healthcare providers. To enhance adherence and improve patient outcomes, healthcare providers should focus on delivering clear, comprehensive information and fostering strong support systems for patients.

## Graphical abstract

**Figure:**
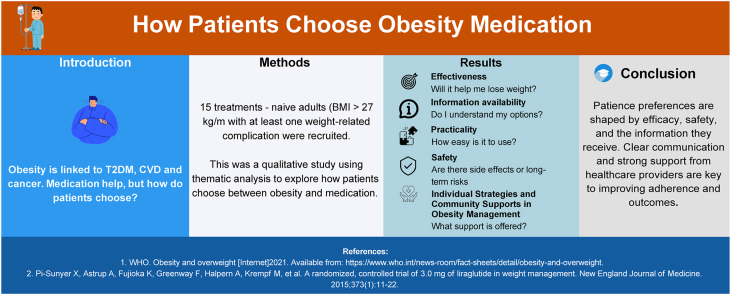

## 1 Introduction

Obesity is a global epidemic with the World Health Organization (WHO) estimating that more than 650 million adults were living with obesity in 2020 [1]. The rising prevalence of obesity is of particular concern due to its strong association with a range of serious comorbidities, including type 2 diabetes mellitus, cardiovascular diseases, and certain types of cancer. These conditions significantly contribute to global morbidity and mortality, while placing immense financial strain on healthcare systems. As a result, effective strategies for managing obesity have become a central focus of public health initiatives worldwide. Obesity medications (OM) have emerged as an effective treatment for the disease of obesity. OM encompasses a range of medications designed to target key physiological mechanisms, such as adipocyte mass control, appetite, and reduced absorption of dietary fats [2].

Despite the demonstrated clinical efficacy of OM in randomized controlled trials, real-world adherence to these medications remains low. Many patients discontinue treatment prematurely due to a combination of factors, including concerns about side effects, perceived lack of effectiveness, and uncertainty about the long-term safety of these medications [3]. These challenges underscore the importance of understanding the factors that influence patient preferences for OM. By identifying the determinants of patient decision-making, healthcare providers can better tailor treatment plans to address patient concerns, improve adherence, and ultimately enhance treatment outcomes.

The *Stratification of Obesity Phenotypes to Optimize Future Obesity Therapy (SOPHIA)* project is a multinational, multidisciplinary initiative committed to improving clinical management of obesity through targeted, evidence-driven interventions. Previous SOPHIA investigations have provided an essential foundation for understanding how individuals living with overweight or obesity navigate diverse treatment pathways, emphasizing the significance of tailored care and robust patient-provider communication [4,5]. These studies uncovered recurring themes—such as the interplay of treatment effectiveness, safety concerns, and patients’ beliefs about their own health—that collectively shape treatment decisions.

However, while early SOPHIA research touched on medication use, there remained a critical gap in our understanding of how people specifically evaluate and select from the expanding array of pharmacological options now available. Recent qualitative findings indicate that individuals weigh not only a drug's capacity to reduce weight or address complications (like type 2 diabetes or hypertension), but also a host of more personal factors such as tolerability, convenience, affordability, and perceived risk. To extend these insights, our current study focuses squarely on patient preferences for obesity medication pharmacotherapy.

Although extensive research has examined the clinical outcomes of OM, there has been relatively little focus on the subjective factors that influence patients' preferences for one treatment above another. Understanding the psychological, social, and contextual factors that shape patient decision-making is essential for developing patient-centered approaches to obesity management. Previous studies have highlighted that patients’ adherence to pharmacotherapy is not driven solely by clinical efficacy but is also influenced by individual perceptions of the risks and benefits, health literacy, and the availability of support from healthcare providers and family members [6,7]. This study explored the factors that guided patient preferences for OM to fill the knowledge gap.

## 2 Methods

### 2.1 Study design

Two of the authors employed a qualitative research design using reflective thematic analysis to explore patient preferences for OM due to its ability to capture the depth and complexity of individuals' experiences and preferences, particularly in relation to their personal health and decision-making processes. Thematic analysis was conducted both inductively (allowed themes to emerge from the data itself) and deductively (guided by pre-existing theoretical frameworks, such as the Health Belief Model [6].

### 2.2 Participant recruitment

15 potential participants were identified and approached by healthcare professionals at an obesity clinic in Dublin, Ireland. The study used purposive sampling, a non-probabilistic sampling technique that involved selecting participants based on specific inclusion criteria that aligned with the research objectives. After providing informed consent, participants were contacted by the research team to schedule an interview. Data collection occurred between June and August 2024 and the ethical approval was obtained from University College Dublin (Reference number: LS-23-74-LeRoux).

The inclusion criteria for participants were: Age between 18 and 70 years; Body mass index (BMI) ≥27 kg/m^2^ with at least one weight-related complication (e.g., type 2 diabetes, hypertension, or dyslipidemia) and treatment-naïve to OM, meaning they had not previously taken medications specifically prescribed for obesity. Participants were excluded if they had severe psychiatric disorders, were planning or had undergone bariatric surgery, or were currently pregnant or breastfeeding. Participation in the study was entirely voluntary, and no incentives or payments were provided to participants for their time.

### 2.3 Data collection

Semi-structured interviews were the primary method of data collection. Each interview lasted between 30 and 60 min 15 interviews were conducted via Zoom. Before the interviews, participants were shown a 10-min informational video that provided a standardized overview of the pharmacotherapy options for obesity treatment available in Europe now and in late-stage development. The medication options discussed included Orlistat, Naltexone/Buproprion, Semaglutide, Tirzepatide, CagriSema, Survodutide, and Retatrutide. The video covered the mechanisms of action for different medications, potential benefits as proven by randomized controlled trials in 2024, side effects, and expected outcomes. Patients were, however, informed before viewing the video that they should consider a situation where the medications are free and readily available.

The interview guide explored a range of emerging themes, capturing diverse perspectives on treatment experiences. Key areas of focus included participants views on treatment effectiveness, their concerns regarding safety and potential side effects, and the influence of healthcare providers in their decision-making. The analysis aimed to remain open to unexpected insights, allowing the data to shape the identification of relevant themes.

All interviews were audio-recorded by the main author with participants' consent and transcribed verbatim. Additionally, field notes were taken during the interviews to capture any non-verbal cues or contextual details that could provide further insights into the participants’ experiences.

### 2.4 Data analysis

The 15 transcripts were anonymized and were input in the MAXQDA 2024 software by two independent coders for analysis using the Braun and Clarke's six-phase thematic analysis framework [8] which included: (i) familiarization of the data, (ii) generating initial codes, (iii) searching for themes, (iv) reviewing themes, (v) defining and naming themes and (vi) producing the report. Data was interpreted to explore and understand the determinants of patient preferences in OM as well as the factors that guided patient preferences for OM. To enhance analytic rigor, a second researcher independently coded all interview transcripts. The two coding frameworks were compared, and discrepancies were resolved through team discussion until agreement was reached on a harmonized set of themes and sub-themes, thereby increasing the validity and credibility of the findings.

## 3 Results

### 3.1 Study participants socio-demographics

Table 1 presents a summary of data collected from 15 respondents regarding their gender, Body Mass Index (BMI) and comorbidities. The BMI values indicate a range of obesity levels with the highest recorded being 53.9 and the lowest at 30.0. Several respondents reported multiple comorbidities indicating a correlation between higher BMI and the prevalence of health issues.

**Table 1:** Demographic information of participants (n = 15).

Respondent	Gender	BMI	Comorbidities
1	Male	46.0	MASLD (fatty liver), Sleep apnoea
2	Male	37.0	Nil
3	Female	51.0	Hypertension, Mixed anxiety & depression, PCOS, ADHD, Autism
4	Female	53.9	Nil
5	Female	37.72	Hypertension, Anxiety
6	Female	37.0	Hypertension
7	Female	33.8	Hypothyroidism, Anxiety, Depression
8	Male	41.0	Dyslipidemia, Gout
9	Female	33.3	Endometriosis, Hypothyroidism
10	Male	37.0	Dyslipidemia, Asthma
11	Female	39.1	Graves' Disease, Fibromyalgia, PTSD/Anxiety
12	Female	nan	Did not attend
13	Female	30.0	Depression, Dyslipidemia
14	Female	36.0	Hypertension, Hypothyroidism, Dyslipidemia, Mild Depression
15	Male	36.5	Impaired Blood sugar

### 3.2 Themes and sub-themes

The purpose of this study was to investigate the determinants that guided treatment preferences among individuals living with overweight or obesity. The themes that emerged from the data were Effectiveness of medication, Information to make decisions, Safety of medications, Practicality and Individual Strategies and Community Supports in Obesity Managements as in Table 2.

**Table 2:** Summary of themes and sub-themes.

Themes	Sub-themes	Patient quotes
**Theme 1: Effectiveness of medications**	Weight loss	*“… the last 2 you mentioned to me sounded great because there was a 25 % overall weight loss to the body. So, I'd like the 25 % weight loss to help with health and everything,” (Respondent 12)*
		*“… and also, the depiction that there's actually a graph, we can actually predict that you can loose 10 % of your weight when you use one medication, 20 % of the weight loss and they are replicable. That that was quite exciting.” (Respondent 15)*
	Impact of complications	*“At the moment I've been diagnosed with high blood pressure for the last, probably four and a half years, which I'm on medication for.” (Respondent 5)*
		*“I suffer from gout.” (Respondent 8)*
		*“I have lymphedema.” (Respondent 12)*
	Food noise	*“It would probably be the reduction of food noise would be my main one*.*” (Respondent 4)*
	Efficacy	*“I think, like the more modern drugs, probably they are way more efficient,” (Respondent 1)*
	Health related quality of life	
	Emotional impact	*“I'm not happy with now, and I don't like you know, photographs of myself or anything. I'd be very critical of myself when I would see pictures of me or videos, I would actually avoid them.” (Respondent 6)*
	Body image	*“You know it's the small things when it comes to. you know, worrying about whether you're going to fit in the aeroplane seats and wondering what people think of you when you're eating your dinner at a restaurant. And you know, I think that's constantly in my mind, and it just it kind of builds up over time. And I feel like. If it builds up into such a thing that I just feel like that.” (Respondent 4)*
		*“But anytime a photograph is taken off me. I just oh, I just can't look at it, I think. Oh, my God! I look so bad! I have to do something about myself, and it's my stomach area. My stomach is huge” (Respondent 8)*
	Mental health	*“The problem is how I feel, and how I feel physically is tied into how I feel mentally.” (Respondent 3)*
		*“So, I don't even like to socialize anymore. It's just a block. It's a mental health block as well. The weight just doesn't help, it can be very depressing and it just limits.” (Respondent 12)*
		*“I don't like how I'm feeling. I'm tired all the time, because I know I'm carrying around the extra weight. I don't have a I don't have a positive image all myself.” (Respondent 9)*
**Theme 2: Information to make decisions**	General practitioner	*“… you know weight loss is only going to help that. I've been told that many times by doctors and my GP, and I think, yeah, it'll definitely have a huge effect.” (Respondent 4)*
		*“I suppose the first thing I did was speak to my GP. I think that's always a good place to start” (Respondent 5)*
	Social media and friends	*“… the literature is out there, print media and the social media and there is evidence that if you can control your weight, you have benefits in your heart that's really drives it.” (Respondent 15)*
	Health literacy	*“While I'm honest, do I have to have check-in with the doctor or anything? Suppose maybe if there was a lot of that involved, it might be a consideration for me.” (Respondent 6)*
		*“But I'm fully prepared to take a chance with this. I feel I've no other choice.” (Respondent 8)*
	Accurate/reliable information	*“It felt very interesting that different medication would have different impacts in relation to diabetes or heart health conditions and stuff like that. So that was very interesting. It's something that probably would factor into the decision making for me, you know.” (Respondent 10)*
		*“I suppose if I was well aware of the promised evidence of other benefits that was mentioned in the video, if those were in it would affect my decision.” (Respondent 14)*
**Theme 3: Safety of medications**	Safety profile	*“If there is proof and evidence that the medication has been around for a while and has shown a safety profile definitely that will be one of the reasons I'll consider before picking up a medication” (Respondent 15)*
		*“If they had satisfactory safety data … but it had a better effect on my health. I might be more likely to go for that one simply because I've tried one of them before” (Respondent 6)*
	Side effects	*“They all have similar side effects in terms of nausea” (Respondent 2)*
		*“I also have high cholesterol as well and I know that medication is the next thing for me.” (Respondent 7)*
		*"I've also had gestational diabetes in a previous pregnancy. So, I suppose. Yeah, I'm just looking at ways to safeguard my future health.” (Respondent 5)*
**Theme 4: Practicality**	Ease of use: Injection Vs Oral tablets	*“I think it has kind of changed my mind. I was quite attracted to the pill form of medication rather than the self-injections, just because it seems a little bit less invasive.” (Respondent 4)*
		*“Initially. people had to go through surgery to get a hold of them of their weight. But now, with a flurry of information that actually they can be an intervention uh and this can be medical, and for some of them you can actually get an injection weekly and the results from these medications are quite impressive.” (Respondent 15)*
	Affordability	*“I don't think I would go just for the cheaper itself, but more like I will set a budget and based on what I can't spend. I will try to not go over that” (Respondent 1)*
		*“I would probably opt for the cheaper one” (Respondent 13)*
	Availability	*“So, I think if it's like available more widely available, I think that that would be would be better for me.” (Respondent 1)*
		*“there's obviously the availability part in terms of it actually being like the pharmacist being able to issue the prescriptions, but also, I guess, the ease of getting medication actually prescribed by a doctor would probably impact things as well” (Respondent 3)*
**Theme 5: Individual Strategies and Community Supports in Obesity Management**	Proactive Health Planning	*“I think research is very important. I think you've got to kind of look for what is going to benefit you the most and what kind of side effects you'd be willing to put up with for a little while” (Respondent 4)*
		*“Do your own research on it as well, and like, you know I would advise somebody. don't go into this blindly. Be very aware of the side effects of all medications” (Respondent 8)*
	Community Influence and Peer Support	*“… like tackle with medications that help like a more aggressive weight loss and then. once you have, like a higher range of motion, and you have more mobility, like introducing exercise and the health style combination as well.” (Respondent 1)*

#### 3.2.1 Effectiveness of medications

In the below quotes, the study participants stated that their choice of obesity medication is partly determined by the weight loss, impact on complications and the health-related quality of life that affected their functionality, mental health, food noise as well as efficacy.

##### 3.2.1.1 Weight loss

The study participants articulated several additional benefits they considered when evaluating the use of obesity medications, extending beyond mere weight loss. In terms of weight loss expectations, participants specified target percentages of 15 %–25 % as quoted below:

*“I would probably be looking at around 15 % ideally.” (Respondent 4)*

*“… definitely like 25 % is more appealing.” (Respondent 1)*

The overall success of the medication in achieving weight loss was a critical factor for participants. One respondent explicitly stated that their primary concern was the drug's ability to facilitate substantial weight loss, underscoring the importance of effectiveness in their decision-making process.

*“for me would be the success of the drug in terms of actually achieving some weight loss.” (Respondent 3)*

Contrastingly, Respondent 5 talked with a nonchalant attitude towards the idea of weight loss indicating that it is not their primary concern. The quote below highlighted a nuanced view of body image and weight where personal priorities take precedence over societal expectations.

*“It's not my primary concern. I mean, it would be nice. I think it will be almost a nice side effect to you know. Be a smaller size, and be able to fit into more clothes, etc." (Respondent 5)*

Respondent 10 also expressed interest in the varying impacts of different medications on conditions such as diabetes and heart health and found it interesting to learn about how various medications can differentially affect health issues.

*“It was very interesting that different medication would have different impacts in relation to diabetes or heart health conditions and stuff like that. So that was very interesting. It's something that probably would factor into the decision making for me.” (Respondent 10)*

##### 3.2.1.2 Impact on complications

Participants were aware of the health complications associated with obesity particularly focusing on complications such as diabetes, dyslipidaemia, and hypertension.

*“So, I think diabetes is one drastic.” (Respondent 2)*

*“I also have high cholesterol as well and I know that medication is the next thing for me.” (Respondent 7)*

*"And of course, being overweight … It's something they will always keep in mind like your blood pressure may be too high, or you know."(Respondent 1)*

##### 3.2.1.3 Food noise

Respondent 4 expresses a sense of optimism regarding the effectiveness of specific weight management strategies noting their positive impact not only on weight loss but also on overall lifestyle improvements.

*“It seems to a couple of the medications that talk about the elimination of food, chatter, or food noise. I think the idea of decreasing or even getting rid of the food noise in the brain is a huge one for me.” (Respondent 4)*

##### 3.2.1.4 Efficacy

The study participants emphasized the importance of selecting a medication that is effective in achieving their desired outcomes. Study participants 11 and 1 clarified that the effectiveness of the obesity medication was fundamental in determining the most appropriate one for their health.

*“I would like something that's effective. I'd rather pay for the more effective drug.” (Respondent 11)*

*“… the more modern drugs, probably they are way more efficient, involving more research and things like this.”****(****Respondent 1)*

##### 3.2.1.5 Health related quality of life

###### 3.2.1.5.1 Emotional impact

This encompassed patient's overall perceptions of how their disease and treatment affect their emotional wellbeing and body image. In this study, participants shared their experiences and feelings related to various aspects of health-related quality of life, particularly focusing on emotional and body image concerns.

*" … you're getting worried about how it can affect you in a negative way. And then you start like gaining weight again and getting frustrated. is a bit scarybecause of the side effects. " (Respondent 1)*

###### 3.2.1.5.2 Body image

Body image emerged as a significant theme affecting participants’ self-perception and confidence. This is explained from the below quotes.

*“I avoid pictures with my kids. I find that sad. So it's really down to mood and my overall health.” (Respondent 7)*

*“I don't like the body image wise. I don't feel like myself.” (Respondent 11)*

###### 3.2.1.5.3 Mental health

The psychological effects of obesity and its treatment were also prominent in participants’ narratives. Many reported experiencing depressions, which they associated with limited mobility and the social isolation that often accompanies it.

*“You get very down when you've limited mobility … I don't even like to socialize anymore … it can be very depressing. It's hard.” (Respondent 12)*

*“My mental health, in a way I have started and failed so many times. I'm starting to feel like I kind of can't really do it on my own,” (Respondent 4)*

In contrast, another respondent emphasized that their weight was not a significant source of mental distress but rather their primary concerns centered around health-related issues such as high blood pressure and a history of gestational diabetes.

*“I mean, my weight is not something that concerns me massively from a mental health perspective. My main concern is health anxieties over like having high blood pressure at my age, and having had a history of gestational diabetes that plays on my mind more than my weight.” (Respondent 5)*

#### 3.2.2 Information to make decisions

##### 3.2.2.1 General Practitioner

Participants in this study underscored the significance of obtaining information from healthcare professionals reflecting a strong appreciation for their expertise and guidance.

*“I’ve been given ample information from different professionals, whether that be dieticians, doctors, kind of online coaches.” (Respondent 4)*

##### 3.2.2.2 Social media and friends

Participants acknowledged the prevalence of information regarding obesity medications on social media and through personal networks. Respondent 5 noted the abundance of discussion about specific drugs like Ozempic on social media as well as anecdotal evidence shared by friends.

*“I know there's a lot of stuff on social media at the moment about different drugs, obviously Ozempic being the main one that I would have heard about through friends.” (Respondent 5)*

##### 3.2.2.3 Health literacy

Based on the findings of the study, the patients were well conversant and had personal reasons as to why they might choose one obesity medication above another. From the quotes below, it was captured that mostly their decision-making process for considering the uptake of obesity medication as a treatment option sparked from a point of struggling with obesity for years and trying all sorts of solutions as well as wanting to loose weight and taking medication that has less side effects.

*“My weight is negatively impacting so I feel like I need extra assistance. I am feeling stuck I have tried … in my situation I just haven't had any luck. So that's why I'm now thinking I just need to maybe bite the bullet and consider medication.” (Respondent 11)*

##### 3.2.2.4 Accurate/reliable information

One respondent emphasized the need to seek out trustworthy sources of information rather than relying on social media or public opinions. Participants indicated that having access to factual, evidence-based information is essential for making informed decisions about their health and the medications they choose to use.

*“So, I think to be transparent is key and an advice for me would be to look on what is out there. Don't look at what's on social media. Don't follow people's opinions.” (Respondent 2)*

Another respondent talked of the importance of informed choices when selecting obesity medications while recognizing that the impact of obesity can vary greatly among individuals. The study participant emphasized on the importance of considering how obesity affects one's day to day life, well-being, mental health, future and family when determining the appropriate course of action. This participant further advocated for making informed decisions that prioritize personal needs and goals rather than being swayed by the experiences of others.

*“I think, put other people's opinions aside like they're not in your situation. They're not going through what you're going through. It affects people differently, you know. It doesn't bother some people when it starts to affect your day-to-day life and your wellbeing and your mental health, your future and your kids, and I think it's something, a decision that you should just go for it … make a decision that is informed” (Respondent 7)*

Another respondent cautioned against rushing into treatment decisions based solely on the experiences of friends emphasizing the need for thorough research and understanding of the available options.

*“Don't go on, your friends have done this, and your friends can get Ozempic and like, and then try and do it all yourself, which I know a lot of people have done. You may get good, but I think in terms of like how to manage it. But the same time you have to understand that it's enough. It's not only that, but it's the overall health benefit of it, but you need to make informed decisions. So, I would say, just do your homework. Don't rush into anything there is support out there” (Respondent 2)*

#### 3.2.3 Safety of medications

##### 3.2.3.1 Safety profile

Participants expressed varying levels of confidence in the safety of the medications. Some respondents indicated a positive perception of safety, expressing their willingness to take the medications due to their perceived safety.

*“I feel they're safe. I’m happy to take them.” (Respondent 9)*

The relationship between safety and the success rates was also evident in the responses. Participants noted that they would be more inclined to choose medications with higher success rates, implying that the perceived effectiveness of a treatment is intertwined with its safety profile. A preference for researched and tried- and-tested medications over newer alternatives was observed among participants.

*“I usually lean towards a tried and tested medication rather than be part of a brand new trial.” (Respondent 10)*

##### 3.2.3.2 Side effects

The responses from participants regarding the side effects of obesity medications revealed a consensus on the common potential side effects associated with their use. A significant number of respondents identified nausea, headaches, gallstones and diarrhea as prevalent adverse effects of these medications.

*“… nausea may be like a frequent symptom of some of those drugs” (Respondent 1)*

*“The headaches, and nausea I think both goes with the drug.” (Respondent 2)*

*“Or any consequences to medication, maybe the risk of gallstones.” (Respondent 2)*

*“upset tummy” (Respondent 7)*

*“I would have diarrhea” (Respondent 3)*

#### 3.2.4 Practicality

The decision-making process regarding the selection of obesity medications was influenced by several key factors, including ease of use, injection versus tablets, affordability and availability.

##### 3.2.4.1 Ease of use: injection versus oral tablets

The quote from respondent 15 reflects a significant shift in perceptions regarding weight management interventions particularly the appeal of medication over surgical options.

“*Initially. people had to go through surgery to get a hold of their of their weight. But now, with a flurry of information that actually they can be an intervention and this can be medical, and for some of them you can actually get an injection weekly and the results from these medications are quite impressive.” (Respondent 15)*

##### 3.2.4.2 Affordability

Several respondents expressed a preference for more affordable treatment options indicating a willingness to accept slower weight loss if it meant accessing cost-effective medications. This is outlined below.

*“I would definitely consider a more affordable option like I don't mind if it takes longer to loose the weight.” (Respondent 7)*

Contrary to the above quote, study participant 6 shared their experience of having spent considerable amounts of money on various weight loss products and slimming clubs without achieving desired results.

*“I spent a lot of money on weight loss products and attended slimming clubs …, if it was good, if it was going to be a better benefit. I think I would pay the money.” (Respondent 6)*

##### 3.2.4.3 Availability

Findings from the study indicated that most of the respondents preferred that the obesity medication is widely and readily available. The two quotes below reported on the same.

*“I'd probably go for what is readily available.” (Respondent 9)*

*“… availability is probably crucial …” (Respondent 2)*

#### 3.2.5 Individual strategies and community supports in obesity management

##### 3.2.5.1 Proactive health planning

The study participants demonstrated a good understanding of obesity management strategies and interventions essential for long-term health planning like doing research, having health checks and joining slimming clubs.

*“I’d advise them to research it thoroughly and have a thorough health check because of heart, and that.” (Respondent 12)*

##### 3.2.5.2 Community influence and Peer support

Respondent 3 noted the influence of community engagement in weight management, observing that many individuals join slimming clubs to achieve their weight loss goals

*“I see other people, you know, and they join slimming clubs, or they do whatever, and they have a little bit of weight to loose.” (Respondent 3)*

## 4 Discussion

The findings of this study provided valuable insights into the complex factors that influenced patient preferences for obesity medications. The five themes identified were 1) Effectiveness of medication, 2) Information to make decisions, 3) Safety of medications, 4) Practicality, and 5) Individual Strategies and Community Supports in Obesity Management which highlighted the multi-dimensional nature of patient decision-making and underscored the importance of addressing both clinical and psychosocial factors in obesity management.

Effectiveness of medications aligned with existing literature on patient decision-making, which emphasized the importance of clinical outcomes in shaping treatment preferences. For patients struggling with obesity, the potential to achieve significant weight loss and improve obesity related complications was a key motivator for initiating pharmacotherapy [7]. There is also sufficient evidence supporting that pharmacotherapy in combination with behavior-based and lifestyle interventions can result in significant weight loss in patients with obesity [9,10]. While some participants hoped for rapid, dramatic results, others had more modest goals focused on improving overall health rather than achieving a specific body weight. Conversely, following weight loss of a patient, weight management was a challenge and behavioral interventions were successful at delivering 5–10 % weight loss change [11]. Likewise, in a previous study [9], it was highlighted that obesity medications cannot be used as a panacea for the treatment of obesity but instead they should be used to facilitate weight control. This variation in expectations in individual strategies and community supports in obesity management that healthcare providers should engage in personalized discussions with patients to clarify realistic outcomes and align treatment goals with individual priorities. Predicting weight loss prior to starting obesity medications are not yet possible and the biology of the disease needs to be carefully explained [12].

Information to make decisions highlighted the critical role of communication in shaping patient preferences and adherence behaviors. Healthcare providers play a central role in offering support by providing regular follow-up appointments, addressing patient concerns, and reinforcing the importance of adherence. Participants who had strong support from healthcare providers and friends reported feeling more motivated to continue with their treatment, even when faced with challenges such as side effects or slow progress. Social networks also contribute by offering encouragement, reminders, and accountability. This finding is consistent with prior research, which has shown that healthcare professionals have a unique and strategically important role in starting the obesity dialogue, performing initial assessment and signposting patients to the most appropriate services [13]. However, patients who lack such support systems may struggle to maintain adherence, highlighting the need for targeted interventions to bolster social support for these individuals. Correspondingly, the engagement between patients and healthcare professionals is fraught with barriers including time limitation, lack of evidence base, sensitivities around raising the topic of obesity with patients and lack of training on the management of obesity [13].

Health literacy played a significant role in shaping participants’ ability to make informed decisions about pharmacotherapy. Participants with higher levels of health literacy were more confident in their decision-making and more likely to seek out additional information from reputable sources. Likewise, individuals with obesity complications reported a higher knowledge of medication and thus may have higher adherence [14]. In contrast, participants with lower health literacy expressed confusion and hesitation about their treatment options, often relying heavily on their healthcare providers to guide their decisions. This indicates that a deficit in health literacy was a challenge in managing obesity. Therefore, reducing deficits in health literacy and social gradients in health literacy should be considered a priority in planning strategies, programs, and activities for the promotion of greater health outcomes [15].

Many participants reported feeling inadequately informed about their treatment options, with some stating that they had to conduct their own research to understand the medications being offered. This lack of clear, accessible information created uncertainty and, in some cases, led to delays in decision-making or outright rejection of obesity medicines. The need for clear, comprehensive communication from healthcare providers is well-documented [16]. Patients are more likely to engage in shared decision-making and adhere to prescribed treatments when they feel informed and confident in their choices. Nevertheless, misinformation among patients about the proper use of medication, recommended diets and disease monitoring can lead to non-adherence [17]. A mixed methods study [18], illustrated that improving access to resources such as specialty clinics and support groups can empower patients with obesity to make informed choices about their treatment.

Safety concerns was a major barrier to initiating or adhering to obesity medication pharmacotherapy. Many participants expressed apprehension about potential side effects, particularly gastrointestinal issues and long-term health risks such as cardiovascular complications. These concerns were compounded by the fact that some participants felt they had not received adequate information from their healthcare providers about the risks and benefits of all the medication choices. Previous studies have similarly found that concerns about safety are one of the most commonly cited reasons for discontinuing pharmacotherapy [18]. In addition, the lack of comprehensive long-term trials raised questions about the safety profiles of the OMs. A challenge still exists in approving obesity medications as there is low availability, low efficacy and adverse effects hence there is a high demand for greater treatment mechanisms and novel drug therapies that are safe and able to control the disease of obesity [11]. Addressing safety concerns was critical for improving adherence to OM. Healthcare providers must take the time to discuss potential side effects in detail, providing clear, evidence-based information about both the short- and long-term risks associated with the medication.

The study participants demonstrated a robust understanding of obesity management strategies, emphasizing the importance of informed decision-making and community support in their health journeys. Their proactive individual strategies included research on treatment options, regular health assessments and participation in social support systems like slimming clubs promoting physical activity, personalized diet and exercise as treatments for the disease of obesity, while obesity medicines and bariatric surgery have even better health outcomes [19,20].

The findings of this study are broadly consistent with existing research on how individuals living with overweight or obesity navigate treatment options, yet they offer a focused examination of patient decision-making around obesity medication pharmacotherapies in particular. Previous investigations underscore the role of comprehensive information, perceived safety, and trust in healthcare providers, emphasizing that people weigh more than just clinical efficacy when determining their treatment pathways [21]. Our results echo those themes but extend this understanding by pinpointing the ways in which individuals specifically compare attributes across different medications.

While earlier work has often included medication alongside surgical and behavioral interventions, the present analysis delves more deeply into how patients prioritize considerations such as medication cost, potential side effects, and daily practicality. In doing so, it expands on the broader frameworks provided by previous studies, which noted the significance of practical barriers but did not systematically track how such factors influence the final choice of a particular drug. Our data show that subtle differences—such as injection frequency, perceived intensity of side effects, and affordability—can be decisive in shaping patient adherence and long-term satisfaction.

In addition to illuminating how patients calibrate the clinical benefits of weight reduction against personal and psychosocial factors, this work offers a more nuanced perspective on the importance of mental health, quality of life, and so-called “food noise.” Although many clinical trials focus on quantifiable markers (such as percentage of weight lost or improved metabolic parameters), our participants explicitly linked medication choice to psychological and social well-being. Their perspectives confirm that, for many individuals, questions of convenience and the ramifications of side effects on daily activities hold as much weight in their decisions as formal measures of drug efficacy.

By systematically mapping these decision processes, the study fills a vital gap between general assessments of patient experiences with obesity treatments and the more granular day-to-day reality of choosing among multiple medication options. This work therefore contributes to a growing body of patient-centered research that emphasizes personalized care, reinforcing the importance of tailoring information and therapeutic choices to address both the medical and lifestyle concerns that shape how people manage overweight or obesity. Such insight can guide clinicians in engaging in more constructive conversations and help refine the design of future interventions targeting improved adherence and meaningful weight outcomes.

### 4.1 Strengths and limitations

This study explored how patients choose obesity medications, using thematic analysis to identify key factors influencing decision-making. Recruitment continued until thematic saturation was achieved, which strengthens the reliability of the findings. Another strength of the study is its patient-centered design, which captures real-world views and preferences often overlooked in obesity pharmacotherapy research. The use of qualitative methods allowed for detailed insights into practical factors such as ease of use, route of administration (injection vs. tablets), affordability, and availability—grouped under the broader theme of practicality.

Other strengths include a clearly defined research problem, a well-supported background showing the clinical importance of the topic, and a clearly stated purpose. The study's conclusion highlights meaningful clinical implications and provides opportunities to improve practice by incorporating patient preferences into obesity care.

However, there are several limitations. The study was limited to individuals with internet access, which may have excluded those from lower socioeconomic backgrounds or with limited digital skills. This could reduce the diversity of the sample. Participation was voluntary and no incentives were offered, which may have led to self-selection bias, attracting individuals more motivated or interested in obesity treatment. In addition, the recruitment method may have favored patients who are more actively involved in treatment decisions and have higher health literacy. Although saturation was reached, the small number of participants limits how widely the findings can be applied. The study was also conducted only in Ireland, which may affect the generalizability of the results to other settings. Finally, because this was a cross-sectional study, it could not examine how patient preferences change over time, such as before and after starting obesity medication.

These limitations highlight the need for caution when interpreting the results and suggest important directions for future research to better understand patient decision-making in obesity treatment.

## 5 Conclusion

This is a qualitative analysis investigating how patients select obesity medications for the treatment of obesity. The study provides important insights into the multifactorial nature of patient decision-making. Five key themes emerged from the thematic analysis: effectiveness of medication, access to information to support decision-making, safety concerns, practicality of use, and individual strategies and community supports in obesity management. These findings highlight the complexity of treatment choices and underscore the importance of shared decision-making. Incorporating these factors into clinical discussions prior to initiating pharmacotherapy may improve patient satisfaction, support individualized care, and enhance long-term adherence to treatment.

## 6 Key takeaway messages

• In a situation where the medications are described as being free and readily available, patient preferences for obesity medications are shaped by treatment efficacy, safety and information provided by healthcare providers.
• Safety concerns regarding side effects and long-terms risks were perceived as major barriers to initiating or adhering to pharmacotherapy
• Healthcare providers should focus on delivering clear, comprehensive information and fostering strong support systems for patients to enhance adherence and improve patient outcomes.

## Ethics statement

This human study was approved by Human Research Ethics Committee – Sciences (UCD School of Medicine) with approval number LS-23-74-LeRoux. All adult participants provided written informed consent to participate in this study. Written informed consent was obtained from the individual(s) for publication of the details of their medical case.

## Author contributions

**(AM) Alvin Mondoh:** Conceptualization, Methodology, Investigation, Data Analysis, Writing - Original Draft, Project Administration.

**(CW) Carel W. le Roux:** Supervision, Conceptualization, Funding acquisition, Writing - Review & Editing.

**(MC) Michael Crotty:** Supervision, Data Analysis, Writing - Review & Editing.

**(FC) Francisca Contreras:** Data Curation, Writing - Review & Editing.

**(HC) Hilary Craig:** Writing - Review & Editing.

## Data availability statement

The data that supports the findings of this study are not publicly available due to ethical guidelines and participant confidentiality but are available from the corresponding author upon reasonable request.

## Declaration of AI use

The authors declare that they have not used any type of generative artificial intelligence for writing of this manuscript, nor for the creation of images, graphics, tables or their corresponding captions.

## Funding sources

SOPHIA has received funding from the 10.13039/501100010767Innovative Medicines Initiative 2 Joint Undertaking under grant agreement No. 875534. The funding source had no role in the study design, execution, data analysis, manuscript conception, planning, writing, or the decision to publish. All the authors of this paper are part of SOPHIA.

## Declaration of competing interest

The authors have no conflicts of interest to declare.

## Figures and Tables

**Appendix 1- Table 1:** Demographic information of participants (n = 15)

Respondent	Gender	BMI	Comorbidities
1	Male	46.0	MASLD (fatty liver), Sleep apnea
2	Male	37.0	Nil
3	Female	51.0	Hypertension, Mixed anxiety & depression, PCOS, ADHD, Autism
4	Female	53.9	Nil
5	Female	37.72	Hypertension, Anxiety
6	Female	37.0	Hypertension
7	Female	33.8	Hypothyroidism, Anxiety, Depression
8	Male	41.0	Dyslipidemia, Gout
9	Female	33.3	Endometriosis, Hypothyroidism
10	Male	37.0	Dyslipidemia, Asthma
11	Female	39.1	Graves Disease, Fibromyalgia, PTSD/Anxiety
12	Female	nan	Did not attend
13	Female	30.0	Depression, Dyslipidemia
14	Female	36.0	Hypertension, Hypothyroidism, Dyslipidemia, Mild Depression
15	Male	36.5	Impaired Blood sugar

**Appendix 2- Table 2:** Summary of Themes and sub-themes

Themes	Sub-themes	Patient quotes
**Theme 1: Effectiveness of medications**	Weight loss	*“… the last 2 you mentioned to me sounded great because there was a 25 % overall weight loss to the body. So I'd like the 25 % weight loss to help with health and everything,” (Respondent 12)*
		*“… and also the depiction that there's actually a graph, we can actually predict that you can loose 10 % of your weight when you use one medication, 20 % of the weight loss and they are replicable. That that was quite exciting.” (Respondent 15)*
	Impact of complications	*“at the moment I've been diagnosed with high blood pressure for the last, probably four and a half years, which I'm on medication for.” (Respondent 5)*
		*“I suffer from gout.” (Respondent 8)*
		*“I have lymphedema.” (Respondent 12)*
	Food noise	*“it would probably be the reduction of food noise would be my main one. .”(Respondent 4)*
	Efficacy	*“I think, like the more modern drugs, probably they are way more efficient,” (Respondent 1)*
	Health related quality of life	
	Emotional impact	*“I'm not happy with now, and I don't like you know, photographs of myself or anything. I'd be very critical of myself when I would see pictures of me or videos, I would actually avoid them.” (Respondent 6)*
	Body image	*“You know it's the small things when it comes to. you know, worrying about whether you're going to fit in the aeroplane seats and wondering what people think of you when you're eating your dinner at a restaurant. And you know, I think that's constantly in my mind, and it just it kind of builds up over time. And and I feel like. If it builds up into into such a thing that I just feel like that.” (Respondent 4)*
		*“But anytime a photograph is taken off me. I just oh, I I just can't look at it, I think. Oh, my God! I look so bad! I have to do something about myself, and it's my stomach area. My stomach is huge” (Respondent 8)*
	Mental health	*“The problem is how I feel, and how I feel physically is tied into how I feel mentally.” (Respondent 3)*
		*“So I don't even like to socialize anymore. It's just a block. It's a mental health block as well. The weight just doesn't help, it can be very depressing and it just limits.” (Respondent 12)*
		*“I don't like how I'm feeling. I'm tired all the time, because I know I'm carrying around the extra weight. I don't have a I don't have a positive image all myself.” (Respondent 9)*
**Theme 2: Information to make decisions**	General practitioner	*“… you know weight loss is only going to help that. I've been told that many times by doctors and my GP, and I think, yeah, it'll definitely have a huge effect.” (Respondent 4)*
		*“I suppose the first thing I did was speak to my GP. I think that's always a good place to start”(Respondent 5)*
	Social media and friends	*“… the literature is out there, print media and the social media and there is evidence that if you can control your weight you have benefits in your heart that's really drives it.” (Respondent 15)*
	Health literacy	*“While I'm honest, do I have to have check-in with the doctor or anything? Suppose maybe if there was a lot of that involved, it might be a consideration for me.” (Respondent 6)*
		*“But I'm fully prepared to take a chance with this. I feel I've no other choice.”(Respondent 8)*
	Accurate/reliable information	*“It felt very interesting that different medication would have different impacts in relation to diabetes or heart health conditions and stuff like that. So that was very interesting. It's something that probably would factor into the decision making for me, you know.” (Respondent 10)*
		*“I suppose if I was well aware of the promised evidence of other benefits that was mentioned in the video, if those were in it would affect my decision.”(Respondent 14)*
**Theme 3: Safety of medications**	Safety profile	*“If there is proof and evidence that the medication has been around for a while and has shown a safety profile definitely that will be one of the reasons I'll consider before picking up a medication” (Respondent 15)*
		*“If they had satisfactory safety data … but it had a better effect on my health. I might be more likely to go for that one simply because I've tried one of them before” (Respondent 6)*
	Side effects	*“they all have similar side effects in terms of nausea” (Respondent 2)*
		*“I also have high cholesterol as well and I know that medication is the next thing for me .”(Respondent 7)*
		*"I've also had gestational diabetes in a previous pregnancy. So I suppose. Yeah, I'm just looking at ways to safeguard my future health.” (Respondent 5)*
**Theme 4: Practicality**	Ease of use: Injection Vs Oral tablets	*“I think it has kind of changed my mind. I was quite attracted to the pill form of medication rather than the self injections, just because it seems a little bit less invasive.” (Respondent 4)*
		*“Initially. people had to go through surgery to get a hold of their of their weight. But now, with a flurry of information that actually they can be an intervention uh and this can be medical, and for some of them you can actually get an injection weekly and the results from this medications are quite impressive.” (Respondent 15)*
	Affordability	*“I don't think I would go just for the cheaper itself, but more like I will set a budget and based on what I can't spend. I will try to not go over that” (Respondent 1)*
		*“I would probably opt for the cheaper one” (Respondent 13)*
	Availability	*“So I think if it's like available more widely available, I think that that would be would be better for me.”(Respondent 1)*
		*“there's obviously the availability part in terms of it actually being like the pharmacist being able to issue the prescriptions, but also, I guess, the ease of getting medication actually prescribed by a doctor would probably impact things as well” (Respondent 3)*
**Theme 5: Individual Strategies and Community Supports in Obesity Management**	Proactive Health Planning	*“I think research is very important. I think you've got to kind of look for what is going to benefit you the most and what kind of side effects you'd be willing to put up with for a little while” (Respondent 4)*
		*“Do your own research on it as well, and like, you know I would advise somebody. don't go into this blindly. Be very aware of the side effects of all medications” (Respondent 8)*
	Community Influence and Peer Support	*“… like tackle with medications that help like a more aggressive weight loss and then. once you have, like a higher range of motion, and you have more mobility, like introducing exercise and the health style combination as well.” (Respondent 1)*

**Appendix 3:** Interview questions guide

Themes	Interview questions
**General Questions**	1.Can you tell me why you consider obesity medications as a treatment option?
	2.After watching the video on available obesity medications, what are your initial thoughts or feelings about these treatment options?
**Safety**	3. How crucial is the safety profile of an obesity medication to you?
	4. Some drugs have longer term safety data but less weight loss, how does that influence your choice of obesity medication?
**Tolerability**	5. How would potential side effects influence your choice?
**Efficacy**	6. How important is the weight loss of the drug when choosing a treatment?
	7. What level of weight loss would you consider sufficient to continue using a drug after 1 year?
**Additional and non-weight related Benefits:**	8. Besides weight loss, what other health benefits would you look for in an obesity drug?
	9. What is the most important health benefit that would influence your choice of obesity medication?
	10. Some medications have more evidence of reducing heart attacks but they have less weight loss, how does that influence your choice of obesity medication?
**Improved Mental Health**	11. How important is the impact of the drug on improving your mental health?
	12. What specific mental health improvements would influence your choice of obesity medication?
**Body Image**	13. How does the potential improvement in body image affect your decision to choose a particular obesity drug?
**Mobility**	14. How important is improvement in functionality or mobility when choosing an obesity medication?
**Cost**	15. If you had to pay for the medication, would you consider a less effective drug if it were more affordable?
**Availability**	16. How important is the availability of a drug in your decision-making process?
**Conclusion**	17. Are there any other factors not mentioned that you think would influence your choice of obesity drugs?
	18. What advice would you give to someone who is starting to explore obesity drug treatments?

Thank you for your time and insights. Your contributions are valuable and will help us better understand the factors influencing treatment choices among patients living with obesity.

## Abbreviations

**BMI**: Body Mass Index
**OM**: Obesity Medications
**WHO**: World Health Organization
**GP**: General Practitioner
